# Difference in Structure and Electronic Properties of Oxygen Vacancies in *α*-Quartz and *α*-Cristobalite Phases of SiO_2_

**DOI:** 10.3390/ma16041382

**Published:** 2023-02-07

**Authors:** Katherine L. Milton, Thomas R. Durrant, Teofilo Cobos Freire, Alexander L. Shluger

**Affiliations:** 1 London Center for Nanotechnology, University College London, Gower Street, London WC1E 6BT, UK; 2 Department of Physics and Astronomy, University College London, Gower Street, London WC1E 6BT, UK

**Keywords:** silicon dioxide, DFT, cristobalite, defects

## Abstract

α-cristobalite (α-C) is a polymorph of silica, mainly found in space exploration and geochemistry research. Due to similar densities, α-C is often used as a proxy for amorphous SiO${}_{2}$, particularly in computer simulations of SiO${}_{2}$ surfaces and interfaces. However, little is known about the properties of α-C and its basic oxygen defects. Using density functional theory (DFT) simulations we provide a comprehensive report on the properties of perfect structure and oxygen vacancies in α-C. The calculated properties of α-C are compared with those of the better-characterized α-quartz (α-Q). Our results demonstrated that the positively charged O vacancy in α-C is most stable in the dimer configuration, in contrast to α-Q, which favors the puckered configuration. A back-projected configuration was also predicted in both polymorphs. We calculated the optical transition energies and isotropic hyperfine constants for O vacancies in both α-Q and α-C, and compared our findings with the results of previous studies and experiments. This work, thus, offers one of the first in-depth investigations of the properties of oxygen vacancies in α-C.

## 1. Introduction

Oxygen vacancies are among the most basic defects in oxide crystals, and their properties affect and control the performance of oxides in technology. Defects in SiO${}_{2}$ have long been the focus of many-electron calculations using quantum chemistry [1,2,3] and periodic DFT [4,5,6,7,8], due to their importance in numerous applications, including microelectronics, catalysis, fiber optics, and dosimetry. These calculations demonstrated that oxygen vacancies in SiO${}_{2}$ distinguish themselves from other oxides in at least two accounts: (1) the number of different configurations they adopt, and (2) the significant distortions they are capable of exerting on the surrounding lattice. Both of these properties are due to the extraordinary flexibility of the SiO${}_{2}$ network. Oxygen vacancies in silica are best studied experimentally in α-quartz (α-Q) and amorphous SiO${}_{2}$ (a-SiO${}_{2}$), where they have been experimentally shown to be stable under many conditions and have at least two (+1 and 0) accessible charge states [9]. We note that, quite loosely, we denote here many types of silica glass, as well as amorphous silica films, as a-SiO${}_{2}$. Although we are aware of many subtle differences, these do not affect our discussion.

On the other hand, theoretical calculations predicted the existence of five charge states of the O vacancy in α-Q and a-SiO${}_{2}$ (with defect charges ranging +2…0…−2) [7,10,11,12,13], as well as more than eight different geometric configurations for the +1 charge state vacancy in a-SiO${}_{2}$ (see, e.g., [3,8]). When transforming between these charge states and configurations, O vacancies in SiO${}_{2}$ undergo structural changes with atomic displacements often exceeding 0.5 Å [3], in excess of typical displacements in other oxides (0.1–0.3 Å [14]). The abundance of predicted configurations of positively charged vacancies in a-SiO${}_{2}$ results from different short and medium range order environments surrounding oxygen sites, due to disorder.

Quite extraordinarily, SiO${}_{2}$ is one of those materials where some of the experimentally suggested defect configurations have never been verified theoretically (e.g., the so called $E_{\delta }^{\prime }$ center in a-SiO${}_{2}$[15]) and a large number of theoretically predicted defect configurations and charge states have not yet been identified experimentally. This huge gulf between theory and experiment is, in part, caused by high energies and the instability of some defect configurations, and by difficulties in experimental measurements and interpretation. The uncertainty in the verification of theoretical predictions undermines current attempts to employ machine learning to predict defect structures in solids (see e.g., [16]) and, particularly, in amorphous SiO${}_{2}$ [17].

One of the longest controversies concerns the existence of the so-called “dimer” configuration of the +1 charge state of O vacancy in SiO${}_{2}$. This simple configuration was predicted by most theoretical calculations of α-Q and a-SiO${}_{2}$ but never observed experimentally, despite numerous attempts (see an overview in ref. [15]). This is likely due to the small barrier for its transformation into the more complex dangling bond configuration of O vacancy, also known as the “puckered” configuration (see details in the next section). Since the existence and stability of both configurations is strongly affected by the short and medium range environment of O sites in SiO${}_{2}$, one could gain additional insights from investigating these defects in other crystalline SiO${}_{2}$ polymorphs. However, surprisingly little is known about the properties of O vacancies in other stable silica polymorphs, such as α-cristobalite (α-C) or tridymite. Samples of these polymorphs are much less abundant and often much more contaminated with impurities than those of α-Q [18,19]. Therefore, theoretical calculations are often the only way to shed light on their properties and provide the information required to identify defects in these polymorphs.

**Figure 1:**
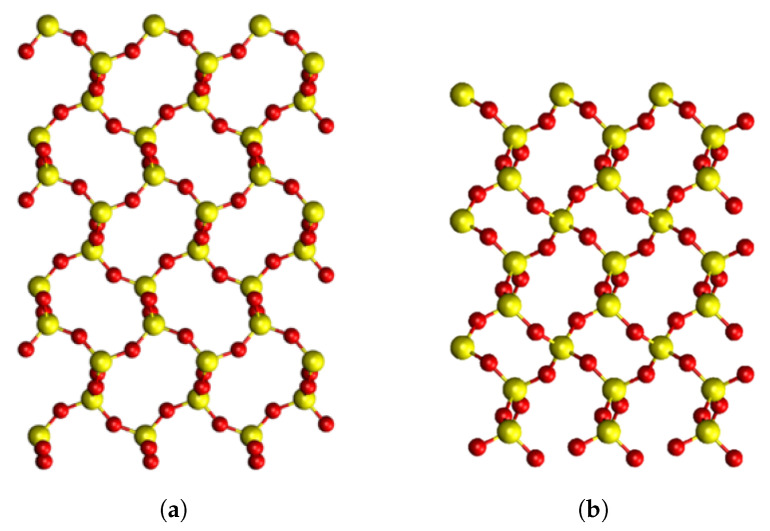
Pristine structures of SiO${}_{2}$ in different phases, viewed along the **a** axis. Yellow atoms correspond to Si, red atoms correspond to oxygen atoms. (**a**) α-cristobalite structure; (**b**) α-quartz structure. Both phases are on the same scale as α-quartz has a smaller unit cell than α-cristobalite.

Here, we focused on calculating properties of O vacancies in α-C, which is a high-temperature and low-pressure silica polymorph. It has a less dense structure than α-Q (see Figure 1) with larger rings close to a-SiO${}_{2}$ structures. There are no theoretical studies of the O vacancies in α-C. By comparing our results for α-C with those for α-Q and a-SiO${}_{2}$ we hoped to better understand the vacancy structure and guide experimental studies of their identification in α-C. In particular, we were interested in comparing the stable configurations of positively charged O vacancies in both crystals.

Our results demonstrated that the positively charged O vacancy in α-C is most stable in the dimer configuration, in contrast to α-Q, which favors the puckered configuration. A back-projected configuration was also predicted in both polymorphs. We calculated the optical transition energies and isotropic hyperfine constants for O vacancies in both α-Q and α-C, and compared our findings with the results of previous studies using molecular clusters [2] and experiments. This work, thus, offers one of the first in-depth looks into the properties of oxygen vacancies in α-C.

## 2. Background

### 2.1. O Vacancy Configurations in SiO${}_{2}$

**Figure 2:**
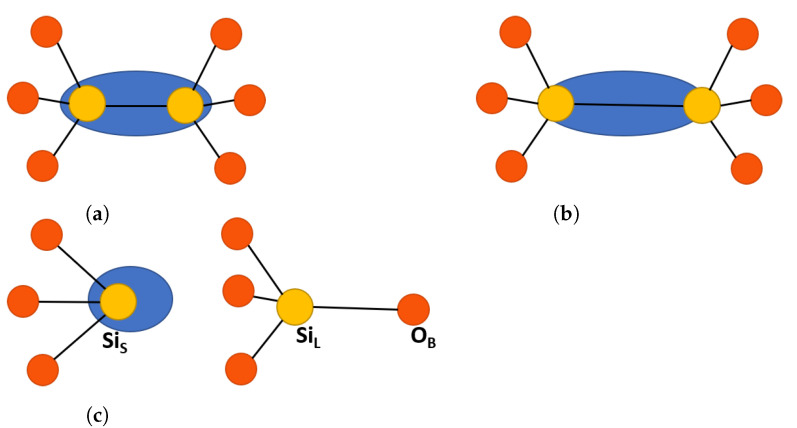
Schematic representation of oxygen vacancy defects in SiO${}_{2}$[8]. Yellow atoms are silicon and red atoms are oxygen, the blue oval represents electron localization. (**a**) Neutral dimer configuration, such as ODC(I). (**b**) Positive dimer configuration, such as $E_{di}^{\prime }$ with elongated Si-Si bonds. (**c**) Puckered $E_{1}^{\prime }$ center, showing the back bond between Si${}_{L}$ and the back oxygen in the surrounding ring, O${}_{B}$. The labeling is consistent with α-quartz and is described in the text.

Previous calculations demonstrated that a neutral O vacancy in α-Q and a-SiO${}_{2}$ (in a-SiO${}_{2}$ this defect is also called the Oxygen Deficiency Center (ODC) I [9,20]) is stabilized by forming a bond between two Si atoms that share two dangling electrons O${}_{3}$≡Si-Si≡O${}_{3}$, or by partially, or fully, passivating these bonds with hydrogen atoms O${}_{3}$≡Si-H H-Si≡O${}_{3}$ [5]. The formation of a bare vacancy in α-Q was accompanied by a very strong displacement of the two Si atoms toward each other, see Figure 2a for the structure of this defect [3,5]. Some authors also suggested the existence of so-called unrelaxed neutral O vacancy in amorphous silica [21]. Hole trapping by neutral O vacancy, or hydrogen de-passivation reactions, can produce positively charged and paramagnetically active defect designated $E^{\prime }$ centers, based on their EPR signals [22,23]. In α-Q, the most stable $E^{\prime }$ center was the $E_{1}^{\prime }$, which was a close relative to the family of $E_{\gamma }^{\prime }$ centers in a-SiO_2_, the general structure of which can be seen in Figure 2c. The $E^{\prime }$ center naming convention uses Greek letters for a-SiO_2_ and Arabic numerals for α-Q. Recent electron paramagnetic resonance (EPR) studies distinguished at least 15 different EPR signals attributed to $E^{\prime }$ centers in different types of α-Q perturbed by different defects and impurities [23,24,25].

Theoretical calculations predicted the existence of two lowest energy relaxed structures of $E_{1}^{\prime }$ center in pure α-Q: the so-called dimer, $E_{di}^{\prime }$, and puckered, $E_{puck}^{\prime }$, configurations [1,10], see Figure 2 and a detailed description below. More *E*′ center configurations in a-SiO${}_{2}$ have been proposed in e.g., references [8,26]. The dimer and puckered configurations differ strongly in terms of the values of isotropic hyperfine constants. The dimer type configurations are characterized by similar hyperfine constants on the two Si ions neighboring the vacancy of around 10 mT. Due to the similarity with observed hyperfine constants, in some papers this configuration has been attributed to the $E_{\delta }^{\prime }$ center [3,5]. However, a thorough analysis of the experimental EPR spectra of the $E_{\delta }^{\prime }$ center suggested a much more complex configuration for this defect involving four or five Si atoms [15,27]. In the dangling bond puckered configuration, the spin density is almost completely localized on one Si ion, which warrants a very strong hyperfine interaction with the Si of 42 mT, close to the measured experimental value [5,24].

In α-Q, the puckered configuration of the $E^{\prime }$ center was shown to be more stable than the dimer configuration in several quite different calculations [5,28,29], supporting the results of EPR measurements [22]. This is related to the structure of α-Q, which has two non-equivalent positions of Si in the crystal structure and, correspondingly, two slightly different Si-O bonds. The so-called Si_S_-O and Si_L_-O bonds, where Si_S_ is the Si atom associated with the shorter bond and Si_L_—with the slightly longer one. The calculations show that the Si_L_ atom can back-bond to O to form the puckered configuration of the $E_{1}^{\prime }$ center, as discussed in more detail below. The most stable configuration of this $E^{\prime }$ center has a forward projection of electrons on the Si${}_{S}$ towards the vacant O site. The less stable $E_{di}^{\prime }$ configuration has not yet been observed experimentally in either α-Q or a-SiO${}_{2}$ [15].

### 2.2. Properties of α-Cristobalite

Tetragonal α-C has a structure similar to α-Q with rings of the same size in both, but α-C has a much lower density (see Figure 1). It is present in various planetary materials, e.g., lunar and martian rocks [30] and meteorites [31]. On the Earth, it occurs as a metastable phase in many geologic settings, for example as crystals deposited from vapor within the pores of volcanic rocks [32]. Most data on this silica phase have been obtained from geochemical analysis of volcanic rocks, where α-C crystals or paracrystals are found with a mixture of silica phases, such as opal-CT [33,34,35]. Extensive studies on the effect of pressure on α-C have contributed to our understanding of the phase [36,37,38,39,40,41,42]. Several earlier computational studies of α-C focused mainly on producing a complete data set of silica polymorphs and lacked detailed characterization of α-C itself [43,44,45,46].

Quite independently of its geochemical and mineral interest, α-C is popular in theoretical calculations of silica as a mimic of the bulk and surface properties of amorphous silica [47,48,49], as both have very similar densities and structural characteristics (e.g., see discussion in [50]). In particular, Emami et al. [51] showed that the α-C (10$\bar{1}$) surface had a similar silanol group density to a-SiO_2_. This makes α-C a good model for the a-SiO_2_ surface structure without the need for computationally demanding melt-quench methods to produce theoretical models of a-SiO_2_ surface structures. Therefore, interfaces of α-C with water [52,53] and with solids have been extensively calculated [48,54,55].

**Figure 3:**
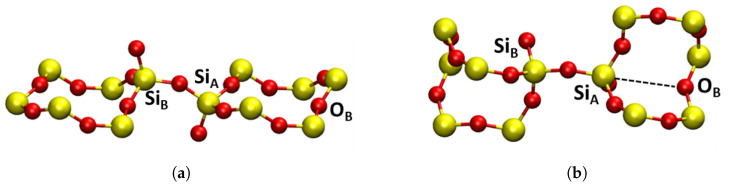
Local pristine structures of (**a**) α-cristobalite with two connected tetrahedrons and (**b**) α-quartz with two connected tetrahedrons with a ring containing back bonding O${}_{B}$. Yellow atoms are silicon and red atoms are oxygen. In α-quartz, Si${}_{A}$ and Si${}_{B}$ are not equivalent, but in α-cristobalite they are equivalent. The Si${}_{A}$ and Si${}_{B}$ notations are, therefore, used to distinguish the Si atoms in defects which are later introduced to the pristine structure. More detail is provided in the text.

In α-C, all Si positions are equivalent, so the distinction of Si_S_ and Si_L_ cannot be made. However, as the *E*′ center is investigated in both, the Si atom involved in the back bonding must be labeled. To distinguish α-C as separate from the α-Q Si_S_ notation, a new labeling was introduced, where Si${}_{A}$ is the Si involved in the back bonding (Si_L_ equivalent) and Si${}_{B}$ is the other Si at the oxygen vacancy (Si_S_ equivalent). For the sake of comparison, α-Q is also labeled in the same way, see Figure 3.

## 3. Materials and Methods

In this paper, we compared properties of O vacancies in two SiO${}_{2}$ polymorphs, α-Q and α-C. Most previous calculations of α-C used molecular cluster models, or local DFT functionals and small periodic cells. In contrast, we used periodic DFT calculations with a hybrid functional and large periodic cells to more accurately predict the structure, electronic, EPR, and optical absorption properties of positively charged, neutral, and negatively charged O vacancies in α-C. These results were compared with those obtained in α-Q and amorphous silica.

Calculations were carried out using DFT implemented in the CP2K software package, using a Gaussian Plane Wave method [56]. A 324 atom 3 × 3 × 3 supercell of α-C and a 260 atom 3 × 3 × 3 supercell of α-Q were used. All calculations were carried out at the Γ-point of the Brillouin zone. Double-ζ Gaussian basis sets [57] were used with the GTH pseudopotential [58]. Plane wave cutoffs were set to 650 Ry (8844 eV) with a relative cutoff of 70 Ry (952 eV). The Broyden–-Fletcher–-Goldfarb–-Shanno algorithm (BFGS) was used to minimize the force on the atoms to 0.0001 a.u (0.005 eV) Å${}^{-1}$ [59].

The local Exchange–Correlation (XC) functional of Perdew–Burke–Ernzerhof (PBE) was used to optimize the cell parameters and atomic positions in all calculations [60]. The optimized structures from the PBE calculations were then used as input for the calculations with the non-local XC functional PBE0–TC–LRC [61]. These PBE0–TC–LRC calculations had an exchange interaction cutoff of 2 Å and a Hartree–Fock exchange of 25%. To reduce the computational cost of non-local functional calculations, the auxiliary density matrix method (ADMM) was used [62]. The same GTH pseudopotentials and plane wave cutoffs were used in both the PBE and PBE0–TC–LRC calculations.

The defect formation energies were calculated using equation [63]:

(1) $$E^{f}\left[X^{q}\right]=E_{tot}\left[X^{q}\right]-E_{tot}\left[bulk\right]+\sum_{i}n_{i}\mu_{i}+q\left(E_{VBM}+\mu_{e}\right)+E_{corr}.$$

Here, $E^{f}\left[X^{q}\right]$ is the defect formation energy, $E_{tot}\left[X^{q}\right]$ is the total energy of the defect system, $E_{tot}\left[bulk\right]$ is the total energy of the pristine system, $\sum_{i}n_{i}\mu_{i}$ is the chemical potential of the element *i* where $n_{i}$ atoms of that type are exchanged with a chemical reservoir, *q* is the defect charge, $E_{VBM}$ is the energy of the valence band maximum, $\mu_{e}$ is the Fermi energy or the chemical potential of electrons, and $E_{corr}$ is a finite-size electrostatic correction using the anisotropic Makov-Payne correction [64,65]. Nudged Elastic Band (NEB) calculations were performed using the Climbing Image NEB (CI–NEB) method [66].

Optical absorption (OA) energies were calculated using the CP2K implementation of the Time-Dependent Density Functional Theory (TD–DFT), which does not include long range correction [67,68]. The truncation cutoff of the PBE0–TC functional was increased, making the function more like the PBE0 XC functional [69]. This was used with an enhanced TZVP–MOLOPT–GTH basis set, as the DZVP–MOLOPT–GTH basis sets did not accurately calculate the optical absorption peak energies. The local structure of the defect was re-optimized with these new parameters.

Positively charged vacancies are paramagnetic and are characterized using Electron Paramagnetic Resonance (EPR). To calculate the hyperfine EPR constants of defects, all-electron calculations were performed using CP2K and a Gaussian and Augmented Plane Wave basis [70]. For these all-electron calculations, pcJ basis sets were employed [71]. The EPR properties were then calculated within the framework of the density functional perturbation theory [72].

## 4. Results of Calculations

### 4.1. Pristine Crystals

First, we compared the properties of α-C and α-Q to understand the underlying structural differences that could contribute to any deviation in the stability of oxygen vacancies. As seen in Table 1, the calculated α-C and α-Q structures in this work were close to the experimental values. The space group of α-C was P4${}_{1}2_{1}2$ with all lattice angles equaling 90. α-Q has a right-hand crystallization P3${}_{2}21$ space group with lattice angles α = β = 90 and γ = 120.

The band gap width of α-Q was studied in more detail than for α-C. The early experimental works by Bart et al. [73,74] showed a range in band gap values in α-Q of 8.6–9.5 eV, depending on the surface structure of α-Q. The bulk experimental band gap values were then determined to be 9.65 eV by Garvie et al. [75,76] A close value was also calculated with high-level theories, such as quasi-particle self-consistent GW [77].

Earlier calculations using the PBE0–TC–LRC XC functional (which was also used in this work) predicted the single-electron α-Q band gap at 8.6 eV [78]. Although this value corresponded well to that by Bart et al. [73,74], it was expected to be an underestimate of the bulk SiO${}_{2}$ value. This underestimate was also seen in this work with a band gap of 8.5 eV.

The band gap values for α-C are only known from theoretical work and depend on the XC functional used. Higher-level XC functionals increase the accuracy of the band gap. The highest level theory calculation found in the literature used a meta–GGA XC functional to produce a single-electron band gap of 8.54 eV, [44]. Consequently, this value was used for comparison. Using the PBE0–TC–LRC XC functional, we calculated the band gap to be 8.57 eV, similar to the meta-GGA functional. Other lower levels of theory produced band gap values in the range of 5.5–10.3 eV [43,45,79,80,81].

The geometry of the two phases show that the Si–O–Si bond angles of α-C are larger than in α-Q. This indicates that α-C has a more open and flexible structure than α-Q, leading to a lower density when compared to α-Q. This is seen in Figure 1, where the large rings in α-C can be clearly seen. In addition, the Si–O bond lengths of α-C were slightly shorter than in α-Q, which might mean that any distortion introduced into α-C introduces more strain as the bonds are perturbed to a greater extent.

The calculated density of α-C (2.35 g/cm^3^) and a-SiO_2_ (2.20 g/cm^3^) [82] showed a difference of 0.15 g/cm^3^, and, thus, the band gaps were expected to differ with a lower density indicating a lower band gap value. However, El-Sayed et al. [83] calculated a band gap value of 8.9 eV for a-SiO_2_ using the hybrid HSE06 XC functional, while experimental band gap values of a-SiO_2_ ranged from 8.9–9.7 eV [75,84,85,86].

In summary, a comparison of the calculated α-Q band gap to the experimental and high-level theory data showed that the α-Q band gap was likely underestimated in this work. Therefore, it was assumed that the α-C band gap was also underestimated. The geometry of the two phases were close to previously observed and calculated results, with α-C having a less dense structure with larger open rings and shorter Si–O bonds.

**Table 1:** The structural properties of α-cristobalite and α-quartz compared to the previous experimental and theoretical literature. The geometric structure is also shown, with the band gap separated at the bottom of the table. All bond lengths and lattice vectors are in Å, all angles are in degrees (°), the density is in g/cm^3^, and the band gap is in eV.

Parameter	α-Cristobalite		α-Quartz	
	This Work	Literature Data	This Work	Literature Data
Lattice Vectors				
*a = b*	5.05	4.97 [87]	4.93	4.91 [88]
*c*	7.08	6.93 [87]	5.43	5.40 [88]
Bond length				
Si${}_{A}$-O	1.604–1.609	1.600–1.607 [87]	1.608	1.604 [88]
Si${}_{B}$-O	1.604–1.609	1.600–1.607 [87]	1.612	1.613 [88]
Bond Angles				
O-Si-O	108–111.8	108.1–111.3 [89]	109.2–110.5	109.0–109.5 [88]
Si-O-Si	150–153	146.6 [87]	144.8–145.1	143.7 [88]
Density	2.35	2.18–2.37 [44,45]	2.41	2.47–2.70 [44,45,90]
Band gap	8.57	8.54 [44]	8.5	9.65 [75]

References [44,45,90] are the results of theoretical calculations. References [75,87,88,89] are experimental data.

### 4.2. Oxygen Vacancies

**Figure 4:**
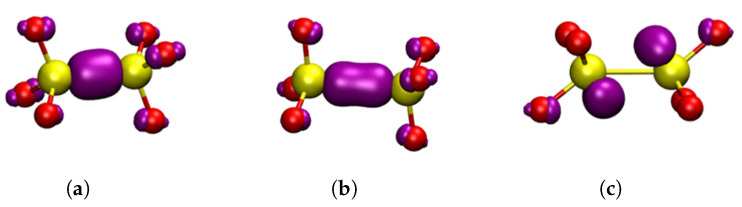
Square modulus of the wavefunction of the highest occupied state in the studied dimer oxygen defects in α-C shown in purple, the isosurface shown is |0.1|. Yellow atoms are silicon and red atoms are oxygen. (**a**) $V_{O}^{0}$ neutral dimer configuration. (**b**) $E_{di}^{\prime }$, positive dimer. (**c**) $V_{O}^{-}$, negative dimer. (**a**–**c**) are general HOMOs applicable to either α-cristobalite or α-quartz.

**Figure 5:**
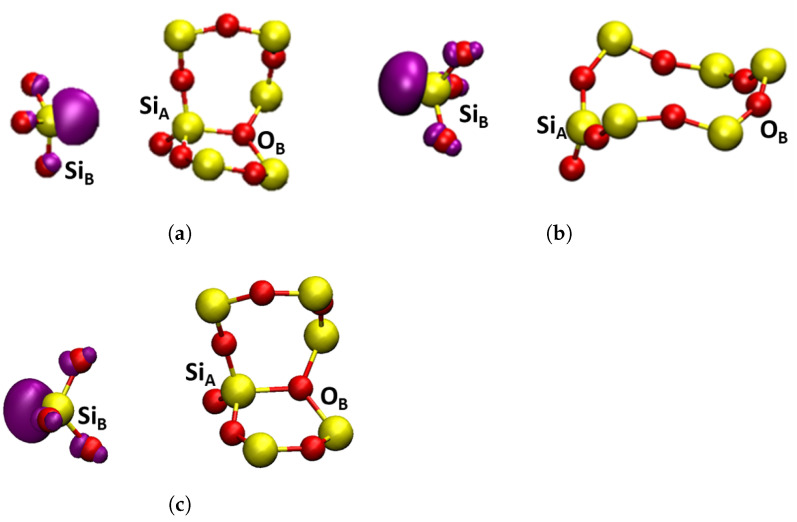
Square modulus of the wavefunction of the highest occupied state in the studied oxygen $E^{\prime }$ defects shown in purple, the isosurface shown is |0.1|. Yellow atoms are silicon and red atoms are oxygen. (**a**) $E_{puck}^{\prime }$, α-quartz puckered, forward projected; (**b**) $E_{bp}^{\prime }$, α-cristobalite, back projected; (**c**) $E_{bp}^{\prime }$, α-quartz, puckered, back projected.

Three charge states of oxygen vacancies were investigated in both α-Q and α-C in the following different configurations: neutral Si-Si dimer ($V_{O}^{0}$), negative Si-Si dimer ($V_{O}^{-}$), positive Si-Si dimer ($E_{di}^{\prime }$), puckered ($E_{puck}^{\prime }$) and back-projected ($E_{bp}^{\prime }$) configurations. The structures and highest occupied molecular orbitals (HOMOs) of these defects are shown in Figure 4 and Figure 5. The short-range geometric parameters of the four configurations are summarized in Table 2 in conjunction with Figure 4 and Figure 5. Below we discuss their geometric, electronic and optical properties in more detail.

#### 4.2.1. Geometric Structure and Stability

##### Neutral Vacancies

Neutral O vacancies in SiO${}_{2}$ are characterized by formation of a Si–Si bond sharing two electrons, which is accompanied by very significant displacement of two Si atoms in the direction of the vacant O site (see Figure 4a and Table 2). Hence, large changes are observed in the Si${}_{A}$-Si${}_{B}$ distance with the introduction of oxygen vacancies in both α-C and α-Q. Compared to pristine structures, the formation of $V_{O}^{0}$ was accompanied by a decrease in the distance between the two Si atoms by 0.81 Å and 0.69 Å for α-C and α-Q, respectively. The character of electron localization between the two Si atoms, forming the bond is seen in Figure 4a.

##### Positively Charged Vacancies

Some of the ways of creating positively charged vacancies in SiO${}_{2}$ are via hole trapping or electronic excitation and ionization of neutral vacancies. Both are complex processes that can lead to significant reorganization of the surrounding lattice due to strong electron–phonon coupling. In static calculations presented here, positive charging of a periodic cell led to hole trapping on the Si–Si bond and the Si${}_{A}$-Si${}_{B}$ distance increased, compared to the $V_{O}^{0}$ defect, by 0.46 Å and 0.59 Å for α-C and α-Q, respectively (see Table 2). If no further reorganization occurs, this configuration of the positively charged defect is called a dimer, $E_{di}^{\prime }$. The Si–Si bond was significantly weakened, due to the loss of an electron and the consequent reduction in bonding order between the two silicon atoms (see Figure 4b), and, due to asymmetry of the lattice structure, more defect configurations are possible.

One such an asymmetric configuration in α-Q has been discovered in references [1,10]. where Si${}_{A}$ in Figure 3b relaxed through the plane of its three back bonded oxygen ions and made a bond with the so-called back oxygen O${}_{B}$, as shown in Figure 5a. This Si atom carried a hole and Si${}_{B}$ carried a strongly localized unpaired electron. This configuration was also predicted to be stable in some sites of a-SiO${}_{2}$ (see discussion in ref. [26]) and is commonly called the puckered configuration. Therefore, we labeled it as $E_{puck}^{\prime }$.

The $E_{puck}^{\prime }$ configuration had the largest Si${}_{A}$-Si${}_{B}$ distance, due to the puckering of Si${}_{A}$ through the plane of O atoms, as seen in Figure 5a, and this distance was close to the literature values [3,5,91]. This configuration was by 0.23 eV lower in energy than $E_{di}^{\prime }$. The CI–NEB calculated adiabatic barrier between the two configurations was 0.26 eV. Similar results were also found in [4,29]. In particular, the results presented in reference [29] demonstrated the dependence of the barrier height on the size of the crystal region allowed to relax. The barrier for the reverse process of conversion from $E_{puck}^{\prime }$ configuration back to the dimer was 0.38 eV, which was in good agreement with previous calculations [5].

The structure of α-C is more symmetric than that of α-Q with Si${}_{A}$ and Si${}_{B}$ being equivalent and O${}_{B}$ further away from them in the corresponding rings (see Figure 3a). Therefore, the $E_{puck}^{\prime }$ configuration proved to be unstable in α-C. However, we found another stable configuration where the Si${}_{B}$, carrying the unpaired electron, was projected through the plane of its three back bonded oxygen ions, as shown in Figure 5b. A similar, so-called “back-projected” configuration, $E_{bp}^{\prime }$, was found in DFT calculations of amorphous SiO${}_{2}$ [8,92]. We note that in this configuration Si${}_{A}$ carrying the hole relaxed into the plane of its nearest oxygen ions. The energy of the $E_{bp}^{\prime }$ configuration was 0.83 eV higher than that of the dimer. The NEB calculation to determine a barrier between $E_{di}^{\prime }$ and $E_{bp}^{\prime }$ did not fully converge, but the barrier exceeded 1.0 eV. These results suggested that $E_{di}^{\prime }$ was the dominant configuration of the positively charged O vacancy in α-C. Below, we calculated its optical and EPR characteristics, which could be used for identifying it experimentally.

The $E_{bp}^{\prime }$ configuration was also stable in α-Q (see Figure 5c), but in this case Si${}_{A}$ formed a bond with the back oxygen O${}_{B}$. Therefore, the energy difference between the more stable $E_{puck}^{\prime }$ and $E_{bp}^{\prime }$ configurations was smaller than in α-C at 0.44 eV. Thus, there were at least three different configurations of positively charged vacancy in α-Q. The calculated EPR and optical properties of the $E_{puck}^{\prime }$ configuration were in agreement with the experimental data for the $E_{1}^{\prime }$ center in α-Q [2,10] (see also our results below) and this configuration was often associated with this defect. Below we compared the calculated optical and EPR parameters of this and other configurations in α-Q and α-C.

##### Negatively Charged Vacancies

The flexibility of the SiO${}_{2}$ structure suggests that the lattice relaxation could promote trapping of an extra electron by a neutral vacancy, as suggested in earlier calculations [10] and, then, further investigated in [11,12]. The results presented here for α-Q and α-C were broadly in line with the previous calculations. The extra electron occupied the anti-bonding state, shown in Figure 4c. The Si${}_{A}$-Si${}_{B}$ distance remained close to that in the neutral vacancy, but the bonds with the nearest O ions were elongated, due to repulsion with the extra electron, as shown in Table 2.

#### 4.2.2. Charge Transition Levels

**Figure 6:**
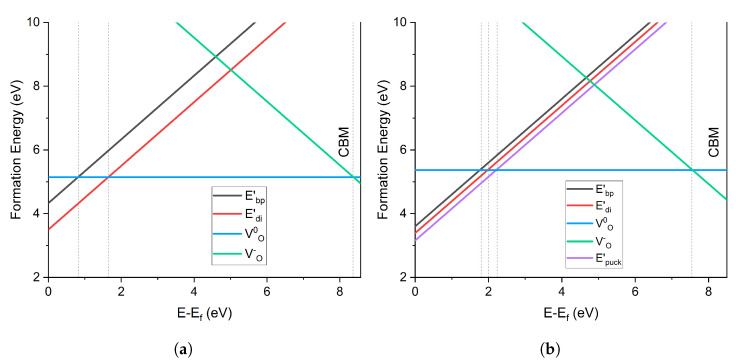
Charge transition level diagram of oxygen vacancy defects in (**a**) α-cristobalite and (**b**) α-quartz. 0 eV is the valence band maximum and the graph cuts off at the conduction band minimum.

The charge transition levels (CTL) of the O vacancy in α-C and α-Q are compared in Figure 6. In α-C, the $E_{di}^{\prime }$ center was the most stable defect at Fermi levels close to the valence band (VB). The transition from $E_{di}^{\prime }$ to $V_{O}^{0}$ (+1 to 0) occurred at 1.64 eV above the VBM, while that from the less stable $E_{bp}^{\prime }$ occurred at 0.81 eV. The transition from $V_{O}^{0}$ to $V_{O}^{-}$ (0 to −1) occurred at 8.34 eV, close to the conduction band minimum (CBM) energy.

On the contrary, the α-Q CTL diagram shows that the $E_{puck}^{\prime }$ configuration was the most stable defect near the VB. The +1/0 transition from $E_{puck}^{\prime }$ to $V_{O}^{0}$ occurred at 2.23 eV above the VBM, whereas the transition from $E_{di}^{\prime }$ occurred at 1.97 eV. $E_{bp}^{\prime }$ was less stable than $E_{di}^{\prime }$, with a transition at 1.79 eV. The negatively charged $V_{O}^{-}$ was more stable in α-Q than in α-C, with a 0/−1 CT at 7.73 eV. The fact that the puckered configuration of the positively charged vacancy was more stable than the dimer configuration agreed with the results of previous calculations in α-Q, e.g., [1,5,10]. However, in α-C positive oxygen vacancies had not been previously investigated, so the contrast in the stability of $E_{di}^{\prime }$ highlights the differences between α-C and α-Q structures.

#### 4.2.3. Optical Absorption

To understand how these oxygen vacancy defects affect the observable properties of the two polymorphs, the optical transitions of α-C and α-Q were investigated using TD–DFT. Due to the CP2K implementation of TD–DFT, the results presented in this section were calculated using the PBE0–TC XC functional, in contrast with the other sections where the long-range GGA correction (LRC) was used. PBE0–TC uses a constant fraction of exact exchange in the short range until a specified cut-off distance, at which it sets exact exchange to zero. To check whether the TD–DFT spectra were sensitive to the cut-off used in truncating the exact exchange, apart from using the 2 Å cut-off, we also used the maximum cut-off for each silica phase, according to the cell size, 6.4 Å for α-Q and 7.5 Å for α-C, respectively. This ensured that the lack of LRC did not introduce artifacts and also meant that the XC functional was almost identical to the classic PBE0 XC functional.

The results shown in Table 3 demonstrate the effect of the cut-off radius on transition energies. We first considered the results for the $E_{puck}^{\prime }$ configuration in α-Q. In Table 3 we only show transitions with oscillator strengths exceeding 0.1. The transitions at 6.18 eV for the 6.4 Å cut-off (and at 6.43 eV for the 2.0 Å cut-off) had a strong contribution from the localized orbitals on the Si${}_{B}$ and atoms in the surrounding rings. The unpaired electron mixed with the Sipd and O*p* states of the surrounding rings, and this formed the bottom of the conduction band. The transition energies were close to the previously calculated values of 5.8–6.1 eV [2,93].

The optical absorption in the 5.8–6.0 eV region in SiO${}_{2}$ was usually associated with the $E^{\prime }$ center and was attributed to its $E_{puck}^{\prime }$ configuration. Experimental optical absorption spectra in α-Q are quite scarce. Guzzi et al. [94] attributed the 6.08 eV peak measured at 77K to the $E_{1}^{\prime }$ center in neutron-irradiated α-Q. Our results at the 6.4 Å cut-off supported this attribution. The nature of electronic excitations responsible for optical absorption of the $E_{puck}^{\prime }$ center was discussed in reference [95] where it was proposed that it was caused by electron transfer from Si${}_{B}$ to Si${}_{A}$ carrying the hole (see Figure 5a). This model was confirmed by DFT calculations in cluster models [2,93]. However, we observed significant contribution of the charge transfer transition only at the small cut-off. The difference in the nature of excitation with the results of [2,93] could be attributed to the much larger size of the periodic cell and more delocalized nature of the CBM states in our calculations, compared to the relatively small clusters used by [2,93]. We observed that optical transition energies were quite robust with respect to the exchange cut-off value and method of calculation.

Similar trends, with respect to the cut-off radius, can be seen also for the $V_{O}^{0}$ center in α-C, where the transition energies with the 6.4 Å radius were reduced by about 0.25 eV with respect to those at 2.0 Å. The main transitions at 7.62 and 7.72 eV were in very good agreement with the absorption peak at 7.6 eV [9,94] usually attributed to the ODC(I) defect, where the proposed structure was a relaxed diamagnetic oxygen vacancy with two electrons localized between two silica [96,97,98] in quartz and SiO${}_{2}$ glass samples. The main transition was between the bonding and anti-bonding σ states localized at the Si–Si bond. The strongly localized nature of the bond might explain why the predicted transitions for α-C are so similar to those in α-Q and a-SiO${}_{2}$, as the long-range structure of the silica might not affect this transition. Two further peaks were seen at 8.2 and 8.48 eV, which could be related to those observed experimentally [94] and were due to localized high-energy π defect states in the conduction band.

The calculated transition energies for the $E_{di}^{\prime }$ configuration in α-C at 7.56 eV and 7.72 eV (see Table 3) overlapped strongly with those for $V_{O}^{0}$ but had lower oscillator strengths. Therefore other data, such as luminescence and EPR spectra, are required for their identification. We noted the similarity with F and F${}^{+}$ centers in MgO and CaO in this respect, where the absorption spectra of these defects also strongly overlapped (see e.g., ref. [99]). Below we present the results of our calculations for the EPR parameters of $E_{di}^{\prime }$ and $E_{puck}^{\prime }$ configurations.

#### 4.2.4. EPR Parameters

The pseudopotentials used so far in this paper were unsuitable for the calculation of EPR parameters, and instead computationally expensive all-electron (AE) calculations had to be performed with an expanded basis set suitable for EPR calculations. Benchmarking calculations demonstrated that, as the quality of the AE basis set was systematically increased, the AE optimized geometry tended to the pseudopotential geometries already obtained. Therefore, the previously calculated defect geometries were maintained, and, then, an AE calculation of the wave function was performed for these geometries. The pcJ basis sets were employed [71], which systematically increased in quality from pcJ-0 to pcJ-2.

The results of these calculations are summarized in Table 4. The isotropic hyperfine coupling constants were calculated for the considered defects, which were dominated by Fermi contact interactions and neglected the subtler anisotropic splittings. As can be seen in Table 4, there were significant differences in the calculated hyperfine couplings between the two configurations $E_{di}^{\prime }$ (in α-C and α-Q) and $E_{puck}^{\prime }$ (in α-Q), due to the differing degree of electronic localization in the two cases. In the $E_{puck}^{\prime }$ configuration, the unpaired electron associated with the defect state was localized on a single Si, and, hence, a strong hyperfine coupling was observed. In contrast, in the $E_{di}^{\prime }$ configuration in both phases, the defect state was delocalized across two Si atoms, and, hence. a much weaker coupling was observed. Although there were important structural differences between α-C and α-Q, our results showed that they did not significantly affect the resultant EPR parameters. The very minor difference in the calculated values for the two Si atoms in the $E_{di}^{\prime }$ defect occurred because the introduction of the oxygen vacancy lowered the symmetry and the two Si sites were no longer symmetrically equivalent.

We note that the hyperfine coupling constant obtained here for the $E_{puck}^{\prime }$ configuration in α-Q is in good agreement with 40.15–42.5 mT attributed to the E${}_{1}^{\prime }$ center [24,100]. These values for the $E_{di}^{\prime }$ configuration are similar to the 10 mT seen in the literature for *E*′_δ_ center [101], as well as in calculations of dimer vacancy configurations [11]. However, the analysis of experimental EPR spectra of the *E*′_δ_ center in silica glass concluded [15,27] that this center had a more complex structure than the $E_{di}^{\prime }$ configuration described here and in previous calculations. Since $E_{di}^{\prime }$ was the only stable configuration of positively charged vacancy in α-C, EPR measurements for the positively charged O vacancy in α-C offered a unique opportunity to identify this configuration and to verify the theoretical predictions.

## 5. Discussion and Conclusions

Predicting properties of defects in solids is a growing activity involving advanced DFT calculations and, increasingly, Machine Learning. Still, the properties of even basic intrinsic defects observed in binary oxides, such as SiO${}_{2}$, are not fully understood. Creating and proving models of defects in solids is still a major challenge. In this work we used the O vacancy in α-quartz to calibrate our computational procedure and test previous predictions using less accurate methods. We then compared the structures and properties of O vacancies in two SiO${}_{2}$ crystalline phases, α-quartz and α-cristobalite, to investigate how structural difference affects these properties and to find ways to identify the dimer configuration of the positively charged O vacancy. The optical absorption spectra of O vacancies in α-C and α-Q were calculated using TD–DFT and isotropic hyperfine constants were calculated for positively charged configurations to aid in experimental identification.

The results for neutral and negatively charged vacancies are similar in both phases and agree with previous calculations. However, there is a significant difference in the number and relative stability of configurations of positively charged vacancies. We found three configurations in α-Q, with $E_{puck}^{\prime }$ being the most stable, followed by the $E_{di}^{\prime }$ and $E_{bp}^{\prime }$ configurations. There were only two stable configurations in α-C, with $E_{di}^{\prime }$ being much more stable than $E_{bp}^{\prime }$. This difference was attributed to the difference in crystal structures and short-range environment of Si atoms in both phases. The back-projected, $E_{bp}^{\prime }$, configuration of the positively charged vacancy was predicted in previous calculations of O vacancies in amorphous SiO${}_{2}$, but had not been considered in crystalline phases. To the best of our knowledge, it has not been attributed, so far, to any experimental signal.

The optical and EPR characteristics calculated for the $E_{puck}^{\prime }$ configuration in α-Q were close to the experimental data for the best-studied $E_{1}^{\prime }$ center. Periodic cells used in our calculations were much larger than 72-atom cells typically used in the past, as well as molecular clusters. They better accounted for the long-range defect-induced lattice distortions predicted by embedded cluster models [3]. However, the optical transition energies were overestimated with respect to the maxima of absorption spectra observed in α-Q (6.08 eV [94]) and a-SiO${}_{2}$ (5.8 eV [9]). TD–DFT showed contributions of both charge-transfer excitations and those into the states more localized on one Si and the surrounding atoms into this absorption peak. We noted that, although our results, in terms of the structure, relative energies, optical and EPR properties were in line with many previous studies of the $E_{puck}^{\prime }$ configuration in α-Q, the accuracy of these calculations, as well as experimental optical measurements, should still be improved to make a fully confident attribution.

On the other hand, both the $E_{di}^{\prime }$ and $E_{bp}^{\prime }$ configurations have never been experimentally identified in either α-Q or a-SiO${}_{2}$. By comparison of hyperfine constants, earlier theoretical simulations associated the $E_{di}^{\prime }$ configuration with the $E_{\delta }^{\prime }$ center observed in a-SiO${}_{2}$[5]. However, several experimental EPR studies ruled out this attribution [15,27]. The predicted stability of the dimer configuration in α-C might provide an opportunity to finally observe and investigate it using the optical transition energies and hyperfine splittings calculated here.

We note that the fact that $E_{di}^{\prime }$ and $E_{bp}^{\prime }$ configurations had not yet been identified experimentally in a-SiO${}_{2}$, in spite of numerous theoretical predictions, may indicate that the O vacancy did not serve as the main precursor for formation of $E^{\prime }$ center. Experiments using field-dependent recombination of holes trapped in thermally grown a-SiO${}_{2}$ films with injected electrons [102] revealed that the paramagnetic state of the $E^{\prime }$ center did not always correlate with the entity bearing the positive charge. It has been suggested that the positive charge is protonic in origin. Consequently, the O${}_{3}$≡Si–H entity in a-SiO${}_{2}$ is suggested to be a possible $E^{\prime }$ center precursor [102], whereupon hole trapping hydrogen dissociates in the form of a proton leaving behind a neutral paramagnetic $E^{\prime }$ center, a hypothesis later corroborated by theoretical calculations [103].

To summarize, these results may help defect characterization in silica phases for geochemistry and space exploration. They demonstrate how subtle differences in the local structure of silica polymorphs strongly affect the structure and stability of basic intrinsic defects. Although the surface of α-C is a good mimic of that for a-SiO${}_{2}$, positively charged O vacancies in the bulk show significant differences in the structure and relative stability of configurations. This may affect modeling of charge transfer processes at interfaces simulated using α-C.

## Figures and Tables

**Table 2:** Bond length (Å) between the atoms surrounding the oxygen vacancy: Si${}_{A}$, Si${}_{B}$, O${}_{B}$ and O surrounding the Si${}_{A/B}$ atoms in α-C and α-Q. For some Si${}_{A/B}$-O bond lengths a range is reported as the bond lengths slightly differ due to asymmetries in the relaxation around the defect. The atoms and labeling conventions are described in Figure 3, Figure 4 and Figure 5. α-cristobalite and α-quartz are compared to understand how they are perturbed between each defect.

Bond	α-Cristobalite					α-Quartz					
	Pristine	$V_{O}^{0}$	$E_{\mathrm{di}}^{\prime }$	$E_{\mathrm{bp}}^{\prime }$	$V_{O}^{-}$	Pristine	$V_{O}^{0}$	$E_{\mathrm{di}}^{\prime }$	$E_{\mathrm{puck}}^{\prime }$	$E_{\mathrm{bp}}^{\prime }$	$V_{O}^{-}$
Si${}_{A}$-Si${}_{B}$	3.11	2.38	2.88	4.59	2.39	3.09	2.41	3.00	4.45	5.38	2.47
Si${}_{A}$-O${}_{B}$	5.31	5.54	5.22	4.83	5.63	3.66	4.10	3.71	1.83	1.84	4.07
Si${}_{A}$-O	1.61	1.61–1.63	1.57–1.58	1.62–1.63	1.67–1.70	1.61	1.63	1.58	1.62	1.61–1.64	1.69–1.73
Si${}_{B}$-O	1.61	1.62–1.63	1.57–1.58	1.54–1.55	1.67–1.71	1.61	1.62	1.57	1.58	1.57–1.59	1.66–1.68

**Table 3:** Optical absorption of oxygen vacancies calculated using different exact exchange cutoff radii. 2 Å is the cutoff used in geometry optimization. The values 6.4 Å and 7.5 Å were the highest cutoff points for the phases of α-quartz and α-cristobalite, respectively. Transition types were determined by the state that had the largest contribution to the excitation. In the spin-polarized calculations, i.e., positively charged defects, the α spin channel contributed to a large extent to the transitions. Transition types are notated by the symmetry of the states, the Si. → Si. indicates a promotion into the singly occupied dangling bond on the same Si atom.

Cutoff Radius (Å)	Phase	Defect	Peak Energy (eV)	Oscillator Strength	Transition Type
7.5	α-C	$V_{O}^{0}$	7.62	0.22	σ→π
			7.72	0.20	
			8.13	0.16	
			8.4	0.13	
		$E_{di}^{\prime }$	7.56	0.12	σ→π
			7.72	0.11	σ→π*
6.4	α-Q	$E_{puck}^{\prime }$	6.18	0.12	Si. → deloc. ring
2	α-C	$V_{O}^{0}$	7.86	0.27	σ→π
			7.94	0.19	
			8.20	0.13	
			8.48	0.12	
		$E_{di}^{\prime }$	6.27	0.15	σ→σ*
			7.86	0.12	σ→π
	α-Q	$E_{puck}^{\prime }$	6.38	0.14	Si${}_{B}$ → Si${}_{A}$
			6.43	0.12	Si. → deloc. ring

**Table 4:** Calculated isotropic hyperfine coupling constants employing the pseudopotential structures and the all-electron wavefunction with the PBE0 functional, as the quality of the pcJ basis sets systematically improved.

AE Basis	α-Quartz (mT)			α-Cristobalite (mT)	
	$E_{\mathrm{di}}^{\prime }$		$E_{\mathrm{puck}}^{\prime }$	$E_{\mathrm{di}}^{\prime }$	
Silica	Si${}_{A}$	Si${}_{B}$	Si${}_{A}$	Si${}_{A}$	Si${}_{B}$
pcJ-0	12.29	10.11	42.25	11.16	10.78
pcJ-1	11.14	8.70	38.96	10.44	10.01
pcJ-2	10.26	8.06	37.46	9.55	9.17

## Data Availability

The data required to reproduce these findings are available from the corresponding author upon reasonable request.

## Abbreviations

The following abbreviations are used in this manuscript:

DFT	Density Functional Theory
α-C	α-cristobalite
α-Q	α-quartz
a-SiO${}_{2}$	Amorphous silicon dioxide
CT	Charge transition
ODC	Oxygen deficient center
EPR	Electron paramagnetic resonance
Opal-CT	Opal cristobalite tridymite
LDA	Local density approximation
GTH	Goedecker-Teter-Hutter
BFGS	Broyden–Fletcher–Goldfarb–Shanno
XC	Exchange-correlation
PBE	Perdew-Burke-Ernzerhof
ADMM	Auxiliary density matrix method
TD-DFT	Time-dependent density functional theory
TC-LRC	Truncated coulomb long-range correction
NEB	Nudged elastic band
CI-NEB	Climbing image nudged elastic band
GGA	Generalized gradient approximation
HSE	Heyd-Scuseria-Ernzerhof
VB	Valence band
CB	Conduction band
CBM	Conduction band minimum
CTL	Charge transition level
TS	Transition state
OA	Optical absorption
AE	all-electron
